# Obesity Alters Gene Expression for GH/IGF-I Axis in Mouse Mammary Fat Pads: Differential Role of Cortistatin and Somatostatin

**DOI:** 10.1371/journal.pone.0120955

**Published:** 2015-03-25

**Authors:** Alicia Villa-Osaba, Manuel D. Gahete, José Córdoba-Chacón, Luis de Lecea, Ana I. Pozo-Salas, Francisco Javier Delgado-Lista, Marina Álvarez-Benito, José López-Miranda, Raúl M. Luque, Justo P. Castaño

**Affiliations:** 1 Department of Cell Biology, Physiology, and Immunology, University of Córdoba, Córdoba, Spain; 2 Instituto Maimónides de Investigación Biomédica de Córdoba (IMIBIC), Reina Sofía University Hospital, Córdoba, Spain; 3 CIBER Fisiopatología de la Obesidad y Nutrición (CIBERobn), Córdoba, Spain; 4 Department of Medicine, University of Illinois at Chicago and Jesse Brown Veteran Affairs Medical Center, Research and Development Division, Chicago, Illinois, United States of America; 5 Department of Psychiatry and Behavioral Sciences, Stanford University School of Medicine, Stanford, CA, United States of America; 6 Lipids and Atherosclerosis Unit, Department of Medicine, Reina Sofía University Hospital, Córdoba, Spain; 7 Mammary Gland Unit, Reina Sofía University Hospital, Córdoba, Spain; Monash University, AUSTRALIA

## Abstract

Locally produced growth hormone (GH) and IGF-I are key factors in the regulation of mammary gland (MG) development and may be important in breast cancer development/progression. Somatostatin (SST) and cortistatin (CORT) regulate GH/IGF-I axis at various levels, but their role in regulating GH/IGF-I in MGs remains unknown. Since obesity alters the expression of these systems in different tissues and is associated to MG (patho) physiology, we sought to investigate the role of SST/CORT in regulating GH/IGF-I system in the MGs of lean and obese mice. Therefore, we analyzed GH/IGF-I as well as SST/CORT and ghrelin systems expression in the mammary fat pads (MFPs) of SST- or CORT-knockout (KO) mice and their respective littermate-controls fed a low-fat (LF) or a high-fat (HF) diet for 16wks. Our results demonstrate that the majority of the components of GH/IGF-I, SST/CORT and ghrelin systems are locally expressed in mouse MFP. Expression of elements of the GH/IGF-I axis was significantly increased in MFPs of HF-fed control mice while lack of endogenous SST partially suppressed, and lack of CORT completely blunted, the up-regulation observed in obese WT-controls. Since SST/CORT are known to exert an inhibitory role on the GH/IGFI axis, the increase in SST/CORT-receptor sst2 expression in MFPs of HF-fed CORT- and SST-KOs together with an elevation on circulating SST in CORT-KOs could explain the differences observed. These results offer new information on the factors (GH/IGF-I axis) involved in the endocrine/metabolic dysregulation of MFPs in obesity, and suggest that CORT is not a mere SST sibling in regulating MG physiology.

## Introduction

The growth hormone (GH) / insulin-like growth factor I (IGF-I) axis plays a crucial role in mammary gland development and breast cancer progression [1–3]. In fact, GH and its receptor (GH-R) as well as IGF-I and its receptor (IGF-IR) have been found to be locally expressed in both mammary gland epithelium and fat pads during all stages of gland development in every mammalian species examined to date [4–7]. Locally produced GH is regulated during mammary gland development, reaching maximum levels in mammary epithelium during puberty (associated to the formation of terminal end buds) but is barely detectable in adult mice [4]. However, locally produced GH does not substitute for circulating GH in terms of promotion of mammary gland development [8]. In this context, the main action of circulating/local GH on the mammary fat pads (MFPs) seems to be stimulation of IGF-I production, as it has been shown that IGF-I mediates all of the actions of GH in mammary gland morphogenesis [2,9,10]. IGF-I, which is locally expressed in MFPs [11] and in mammary gland epithelium [12,13], plays a direct role in ductal morphogenesis and mammary gland development. Indeed, IGF-I knockout mice show impaired development of mammary gland structures [2] and epithelial deletion of IGF-I results in a deficit in ductal branching [12,13]. In contrast to GH, local production of IGF-I in stromal fraction could be more important than circulating IGF-I, at least for ductal development [14]. Nevertheless, regulation of mammary gland physiology by this system is likely more complex, as it interacts with other related peptides, such as prolactin (PRL), and its receptor (PRL-R), which is a well-known key regulatory tandem of mammary gland development and physiology [15].

GH and IGF-I have also been suggested to promote breast carcinogenesis based on several epidemiologic studies indicating that circulating levels of GH and IGF-I are positively correlated with breast cancer risk [16–25] at least, among postmenopausal women [26]. In addition, *in vitro* and *in vivo* studies of rodent and primate model systems show that GH and IGF-I can induce mammary epithelial cell proliferation and differentiation while blocking apoptosis [10]. However, the precise role of the GH/IGF-I axis in breast cancer development and progression is still not fully elucidated, especially in the context of obesity, a metabolic status that is tightly coupled to breast cancer risk, at least, in postmenopausal women [27–29]. The fact that some components of the GH/IGF-I axis are altered in xenographed mammary tumors from obese mice [30] reinforce the crucial role that local GH/IGF-I axis plays in normal and pathologic mammary gland development under different metabolic conditions.

Local expression of GH/IGF-I axis components is regulated in different tissues by circulating and locally produced inhibitory factors, as somatostatin (SST) and cortistatin (CORT), as well as by stimulatory factors, like ghrelin [31–34]. However, the functional relevance and interaction of these factors at the mammary gland level is still unknown. SST was originally discovered by its ability to inhibit pituitary GH secretion [31], but, nowadays it is well established that SST is a multifunctional, pleiotropic hormone that exerts multiple functions through a family of five G-protein coupled receptors (GPCRs) with seven transmembrane domains (TMDs), called sst1-5 [35]. On the other hand, CORT [36,37] is highly similar to SST in structure and sequence, which can explain their common functional abilities, including the inhibition of GH secretion [37,38], and their comparable binding profile to all ssts, which have been shown to be expressed in normal and tumoral mammary gland [39]. Notwithstanding this, CORT and SST show different tissue distribution patterns and exhibit several separate, even opposite, actions [40], likely owing to the ability of CORT to bind additional receptors [i.e. the ghrelin receptor or GH secretagogue receptor (GHS-R)] [41]. Ghrelin, which was identified in stomach as the endogenous ligand of GHS-R [32], can be modified by the ghrelin-O-acyl transferase (GOAT) enzyme with an N-octanoic acid in the hydroxyl group of the third serine residue, an acylation that is critical to exert many of its functions and is essential for binding to its receptor [42,43]. Interestingly, ghrelin is expressed in mammary gland [44], and induces proliferation *in vitro* in breast cancer cell lines [45], however, GHS-R does not seem to be expressed in this tissue [46]. It should be noted that ghrelin gene also encodes different splicing variants, as is the case of the mouse In2-ghrelin and human In1-ghrelin variants [47,48] recently identified by our group, being the expression levels of some of these variants higher than those found for native ghrelin in human breast tumors [48].

In view of the crucial role of GH/IGF-I in mammary gland (patho)physiology, its necessary interdependence with the SST/CORT/ghrelin systems, and the impact that diet-induced obesity (DIO) would likely have in the local expression of these regulatory systems, we hypothesize that a local deregulation of GH/IGF-1 axis (and/or its regulatory systems) may occur in mammary fat pads under obesity conditions and upon the lack of SST or CORT, which could therefore influence the (patho) physiology of the mammary gland. Therefore, the present study was aimed at analyzing, for the first time, the expression pattern of these regulatory systems in the MFPs of CORT and SST knockouts (KO;-/-) in parallel with their respective control mice (+/+) fed a low-fat (LF; lean-control) or a high-fat (HF; obese-group) diets.

## Materials and Methods

### Animals

All experimental procedures were approved by the Animal Care and Use Committees of the University of Cordoba. C57Bl/6J female mice were bred in-house and maintained under standard conditions of light (12-h light, 12-h dark cycle; lights on at 07:00 h) and temperature (22–24°C), with free access to tap water/food.

A set of CORT-/-, SST-/- and their corresponding littermate controls (CORT+/+ and SST+/+) mice generated from heterozygous breeding pairs (n = 12 mice/genotype) were fed a LF diet (*Research Diets*, Gentofte, Denmark; D12450B; 10% Kcal fat, 70% Kcal carbohydrates, 20% Kcal proteins) or HF diet (Research Diets; D12492; 60% Kcal fat, 20% Kcal carbohydrates, 20% Kcal proteins] for 16 weeks, starting at 4 week of age, as previously reported [49]. Mice body weights were monitored twice a week. At least one week prior to killing the mice, all of them were trained and handled in order to acclimate to personnel and handling methods. All females under random cycling condition were killed by decapitation without anesthesia. Trunk blood was collected and inguinal MFPs were harvested using sharp scissors, starting from the proximal area close to the nipple towards the distal end of the gland towards the spine of the animal, collecting all the adipose tissue delimiting the inguinal mammary area. MFPs constitute a heterogeneous population of cells that include adipose stroma and epithelial cells, whose proportions may be modified under obesity conditions. Samples were immediately frozen in liquid nitrogen and stored at -80°C until their further processing.

### Assessment of plasma leptin

Trunk blood was collected from WT, CORT-KO and SST-KO mice after killing and immediately mixed with MiniProtease inhibitor (Roche; Barcelona, Spain), placed on ice, centrifuged and plasma was stored at -80°C until leptin determination. Circulating leptin levels were assessed by using a commercial ELISA kit (Millipore; Madrid, Spain).

### Total RNA isolation and retrotranscription

Total RNA from inguinal MFPs was isolated using TRIzol Reagent (Invitrogen, Barcelona, Spain) following the manufacturer’s instructions and treated with DNase (Promega, Barcelona, Spain). The amount of RNA recovered (before and after DNase treatment) was determined using the NanoDrop2000 spectrophotometer (Thermo Scientific, Wilmington, EEUU). 1 μg of RNA was reverse transcribed to cDNA using random hexamer primers [First Strand Synthesis (MRI Fermentas, Hanover, MD)].

### Quantitative real time PCR (qPCR)

qPCR reactions were performed using the Brilliant III SYBR Green QPCR Master Mix (Stratagene, La Jolla, CA) in the Stratagene Mx3000p system. For each reaction, 10μl of master mix, 0.3μl of each primer, 8.4μl of distilled H_2_O and 1μl of cDNA (50 ng) in a 20μl total volume were mixed. The qPCR was performed with a program consisting of the following steps: (1) 95°C for 3 min, (2) 40 cycles of denaturing (95°C for 20 sec) and annealing/extension (61°C for 20 sec) and (3) a last cycle where final PCR products were subjected to graded temperature-dependent dissociation (55°C to 95°C where it increased 0.5°C/30 sec) to verify that only one product was amplified. Total RNA samples that were not reversed transcribed and a no cDNA control were run on each plate to control for genomic DNA contamination and to monitor potential exogenous contamination, respectively. To control for variations in the amount of RNA used in RT reaction and the efficiency of RT reaction, mRNA copy number of each transcript of interest was adjusted by a normalization factor from two optimum housekeeping genes for mammary fat pads: β-actin and GAPDH, using the Genorm 3.3 visual basic application, where the expression of these housekeeping genes was not significantly altered (data not shown).

Specific primers (Table 1) for mouse transcripts were designed with Primer3 software and validated using the same parameter reported previously [50,51]. Standard curves of each transcript were made and run in parallel with the experimental samples in order to quantify absolute gene expression (copy number).

**Table 1:** Specific set of primers for amplification of mouse transcripts used for quantitative real-time RT-PCR.

*Template*	*GenBank Accession*	*Primer sequence*	*Nucleotide Position*	*Product size (bp)*
**GH**	NM_008117	Sn: CCTCAGCAGGATTTTCACCA As: CTTGAGGATCTGCCCAACAC	Sn 412 As 553	142
**GH-R**	BC075720	Sn: GATTTTACCCCCAGTCCCAGTTC As: GACCCTTCAGTCTTCTCATCCACA	Sn1155 As 1352	198
**IGF-I**	NM_010512.3	Sn: TCGTCTTCACACCTCTTCTACCT As: ACTCATCCACAATGCCTGTCT	Sn31 As 232	202
**IGF-II**	NM_010514	Sn: GCTTGTTGACACGCTTCAGTT As: GAAGTACGGCCTGAGAGGTAGA	Sn 555 As 751	197
**IGF-IR**	NM_010513	Sn: TGGAGTGCTGTATGCTTCTGTG As: CTGGTTTCGGGTTCATCCTT	Sn 3512 As 3691	180
**PRL**	NM_011164.2	Sn: GGCCATCTTGGAGAAGTGTG As: ACAGATTGGCAGAGGCTGAA	Sn 20 As 156	137
**PRL-R**	NM_011169.5	Sn: TGGGAGATCCACTTCACAGG As: GGCCACAATGATCCACACA	Sn 1271 As 1459	189
**SST**	NM_009215.1	Sn: TCTGCATCGTCCTGGCTTT As: CTTGGCCAGTTCCTGTTTCC	Sn 138 As 250	113
**CORT**	NM_007745.3	Sn: AAGAGACCCTCGTCCACCAA As: ACCAGGCAAGGAAAGTCAGAAG	Sn52 As 264	213
**sst1**	NM_009216	Sn: TGCCCTTTCTGGTCACTTCC As: AGCGGTCCACACTAAGCACA	Sn 757 As 891	135
**sst2**	NM_001042606	Sn: CCCATCCTGTACGCCTTCTT As: GTCTCATTCAGCCGGGATTT	Sn 925 As 1058	134
**sst3**	NM_009218.3	Sn: GCCTTCTTCGGCCTCTACTT As: GAATGCGACGTGATGGTCTT	Sn 1292 As 1430	139
**sst4**	NM_009219.3	Sn: AGGCTCGTGCTAATGGTGGT As: GGATGAGGGACACATGGTTG	Sn 860 As 980	121
**sst5**	NM_011425.2	Sn: ACCCCCTGCTCTATGGCTTT As: GCTCTATGGCATCTGCATCCT	Sn1215 As 1319	105
**sst5TMD4**	GQ359775	Sn: GTCCACCCTCTCCGCTCA As: GCAGGTTCGCAGAGGACATC	Sn 415 As 545	131
**sst5TMD2**	GQ359776	Sn: CAGTTCACCCGTACTGTGGCAT As: CACAGCTTCAGGGTGGGTAA	Sn358 As 489	132
**sst5TMD1**	GQ359777	Sn: AACGTGTATATCCAGACAAGAGTGG As: TCCCAGAAGACAACACCACA	Sn 217 As 368	152
**Ghrelin**	NM_021488.4	Sn: TCCAAGAAGCCACCAGCTAA As: AACATCGAAGGGAGCATTGA	Sn163 As 288	126
**In2-Ghrelin**	DO_993169	Sn: GCTGTCTTCAGGCACCATCT As: GTGGCTTCTTGGATTCCTTTC	Sn 1221 As 1444	224
**GOAT**	NM_001126	Sn: ATTTGTGAAGGGAAGGTGGAG As: CAGGAGAGCAGGGAAAAAGAG	Sn 473 As 592	120
**GHS-R**	NM_177330.3	Sn: TCAGGGACCAGAACCACAAA As: CCAGCAGAGGATGAAAGCAA	Sn 1002 As 1072	71
**β-actin**	NM_007393.2	Sn: CTGGGACGACATGGAGAAGA As: ACCAGAGGCATACAGGGACA	Sn 313 As 517	205
**GAPDH**	XM_001473623.1	Sn: ATGGCCTTCCGTGTTCCTAC As: GCCTGCTTCACCACCTTCTT	Sn 757 As 860	104

*Bp* base pairs; *Sn* sense; *As* antisense.

### Statistical analysis

Samples from all groups within an experiment were processed at the same time. 2-way ANOVA was used to compare the influence of the two factors [diet (LF, HF) and/or genotype (WT, CORT-KO, SST-KO)] and their interaction, followed by Bonferroni post-hoc test. All values are expressed as mean ± SEM; p<0.05 was considered significant. All statistical analyses were performed using the GraphPad Prism 5.0 software (GraphPad Software Inc., La Jolla, CA).

## Results

### Effect of HF diet on body weight

**Fig 1:**
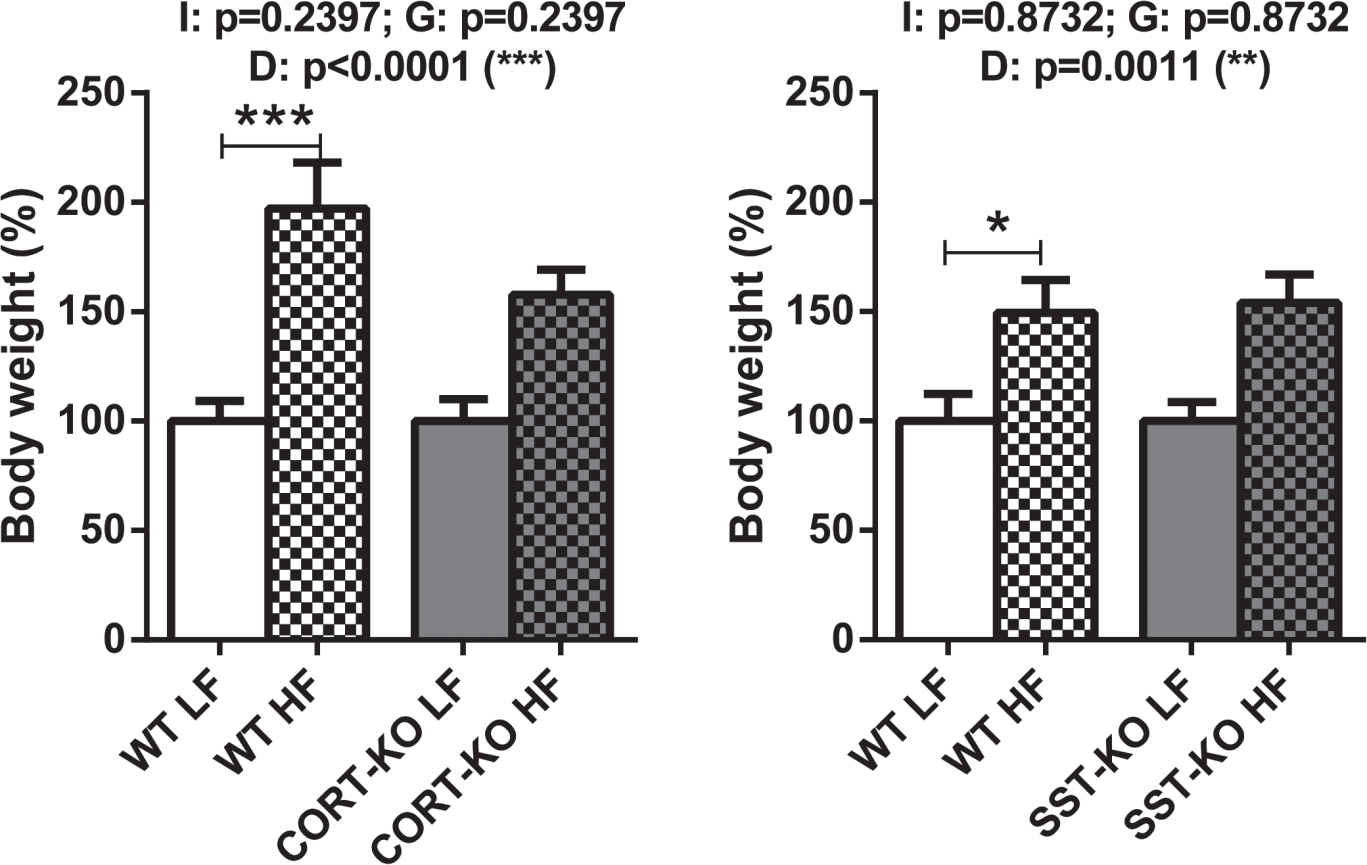
Impact of LF- and HF- diet on body weight in WT, CORT-KO and SST-KO female mice. Values represent percentage of body weight increase compared to 100% of LF diet fed mice. Global differences by 2-way ANOVA are shown at the top of each graphic (G: genotype effect; D: diet effect I: effect of interaction between genotype and diet; *, p<0.05; **, p<0.01; ***, p<0.001). Asterisks above the bars (*, p<0.05; ***, p<0.001) indicate significant differences between groups by Bonferroni post-hoc.

In order to confirm the obese status induced by the HF diet, body weights were recorded at the end of the experiments and percentages of increase in HF-fed animals vs. LF-fed mice are depicted in Fig 1. In CORT group, HF-feeding dramatically augmented the percentage of weight in female WT and CORT-KO mice gain after 16 weeks of diet compared to LF-fed controls. Similarly, in SST group, HF-feeding caused a strong increase in both WT and SST-KO mice body weights compared to LF-fed controls.

### Effect of HF diet on circulating leptin levels

**Fig 2:**
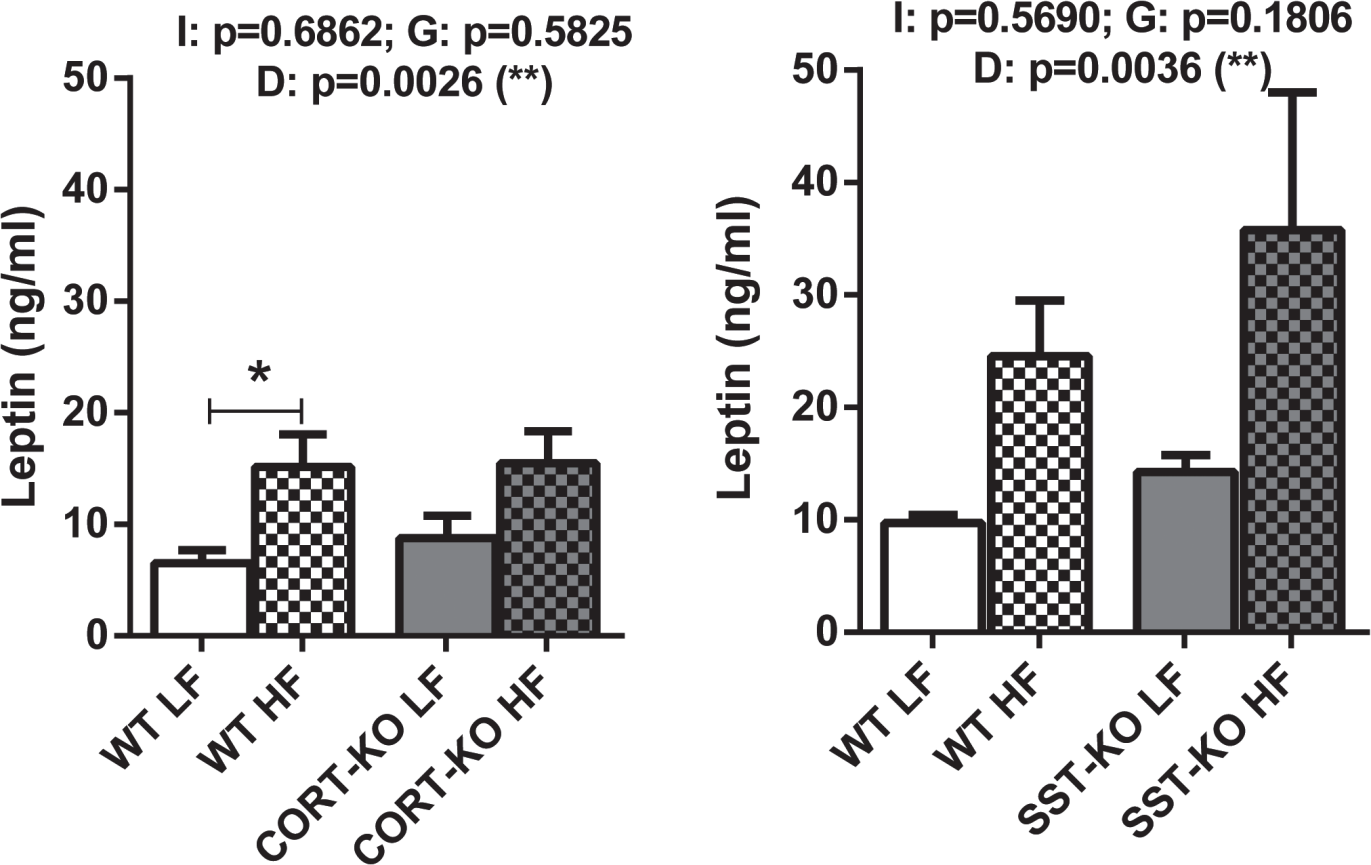
Plasma leptin levels in WT, CORT-KO and SST-KO mice fed under LF and HF diets. Values represent mean ± SEM of ng/ml of plasma leptin. Global differences by 2-way ANOVA are shown at the top of each graphic (G: genotype effect; D: diet effect I: effect of interaction between genotype and diet; *, p<0.05; **, p<0.01; ***, p<0.001). Asterisks above the bars (*, p<0.05) indicate significant differences between groups by Bonferroni post-hoc.

In order to further support the obese status induced by the HF diet, plasma leptin levels were analyzed in SST and CORT groups under LF- and HF- feeding (Fig 2). Importantly, we observed that HF diet caused a significant increase of plasma leptin levels of obese mice compared to lean mice (p = 0.002 in CORT group and p = 0.003 in SST group).

### GH/IGF-I, SST/CORT and ghrelin systems are expressed in mouse mammary fat pads under normal feeding conditions

**Fig 3:**
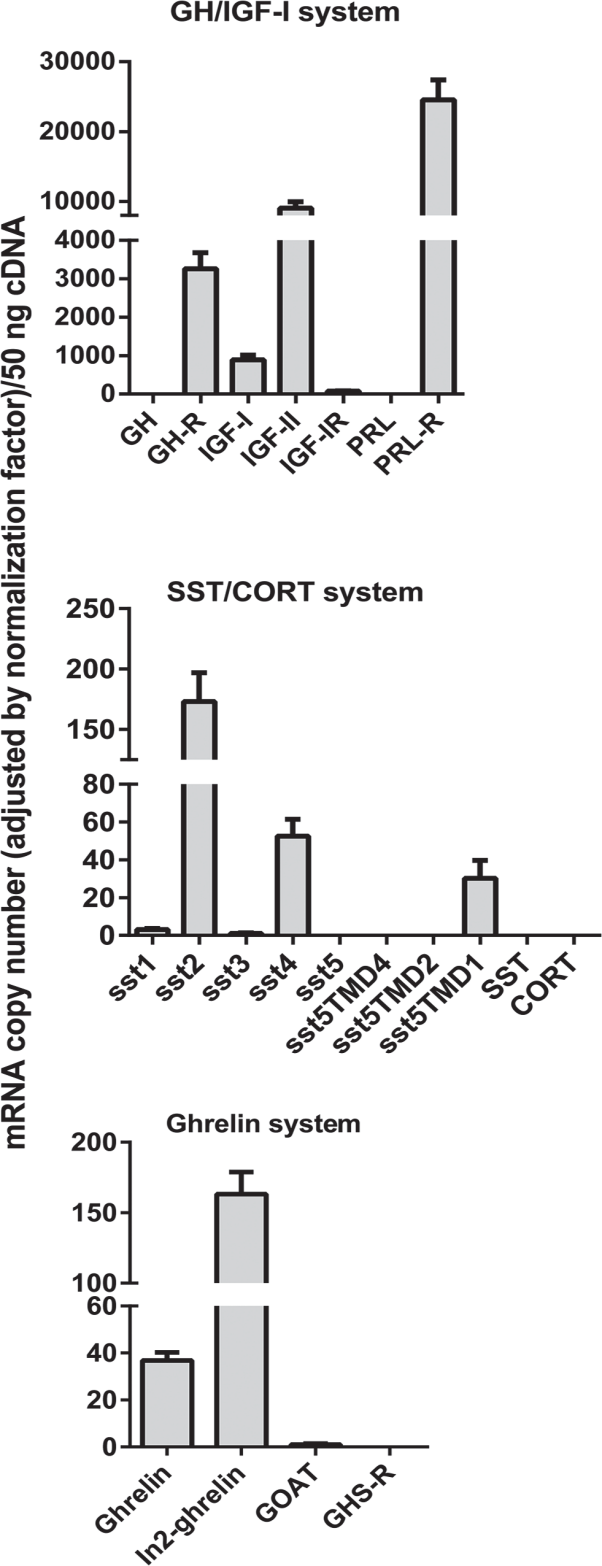
Characterization of GH/IGF-I axis and the regulatory SST/CORT/ghrelin/receptors system in mammary fat pads of female mice (expressed as absolute copy number/50 ng total cDNA). Values represent means ± SEM of the mRNA copy number of each transcript (n = 5–6 mice).

qPCR analysis demonstrated that the components of GH/IGF-I and PRL systems are expressed in MFPs of female mice under normal feeding conditions. GH-R, IGF-I, IGF-II, IGF-IR and PRL-R were expressed at high levels, whereas GH and PRL expression was not detectable (Fig 3, top-panel). The majority of sst subtypes were expressed in mouse MFPs, being their relative abundance sst2>sst4>sst5TMD1>sst1 = sst3 (Fig 3, middle-panel). Conversely, full-length, native sst5 and the ligands (SST and CORT) were not expressed substantially in MFPs. Ghrelin system is also expressed in mouse MFPs, where the In2-ghrelin variant was found to be more expressed than native ghrelin, while GOAT enzyme and ghrelin receptor (GHS-R) were expressed at negligible levels (Fig 3, bottom-panel).

### Impact of DIO and loss of SST/CORT on GH/IGF-I axis in mouse mammary fat pads.

**Fig 4:**
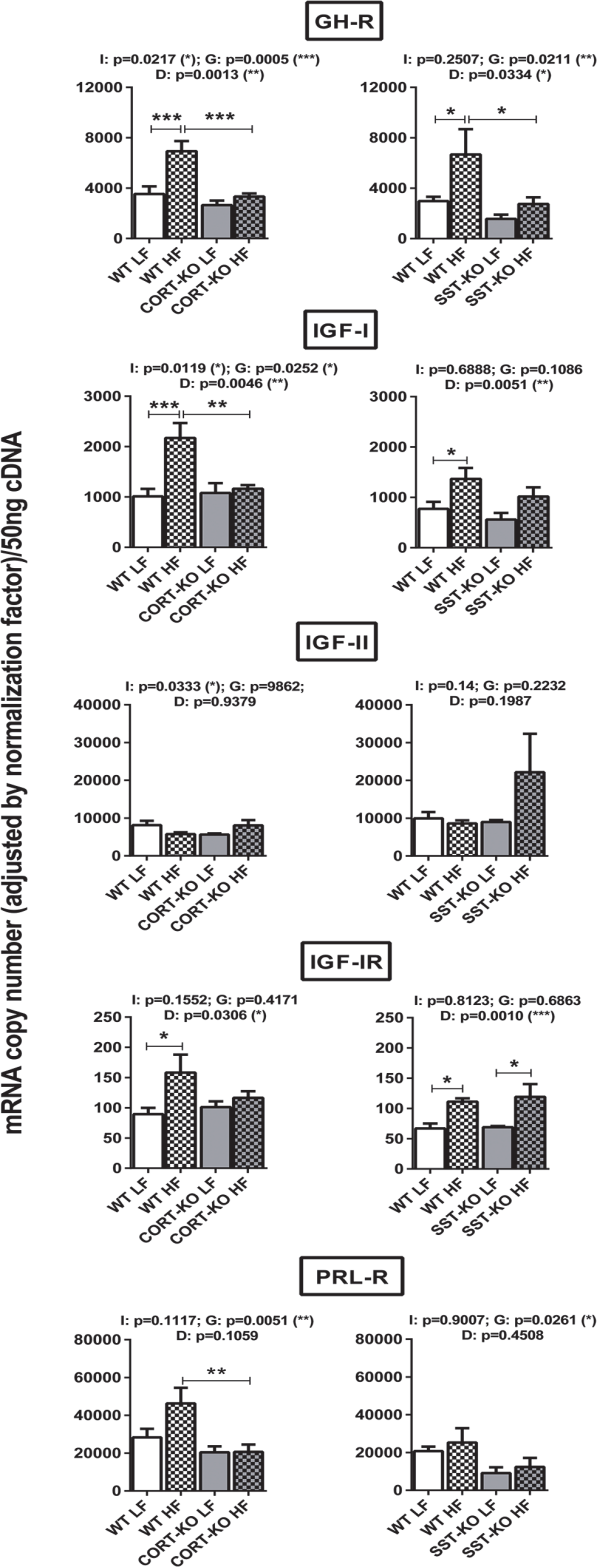
Impact of diet-induced obesity on the expression of GH-IGF-I axis in the mammary fat pads of CORT-KO, SST-KO and their respective control mice. mRNA levels of GH-R, IGF-I, IGF-II, IGF-IR and PRL-R were measured by qPCR. Values represent means ± SEM of the mRNA copy number of each transcript adjusted by the normalization factor (n = 5–6). Global differences by 2-way ANOVA are shown at the top of each graphic (G: genotype effect; D: diet effect I: effect of interaction between genotype and diet; *, p<0.05; **, p<0.01; ***, p<0.001). Asterisks above the bars (*, p<0.05; **, p<0.01; ***, p<0.001) indicate significant differences between groups by Bonferroni post-hoc.

To analyze the effect of diet-induced obesity and the implication of SST and CORT in the regulation of GH/IGF-I, SST/CORT and ghrelin systems expression in the MFPs, we used CORT- and SST-KO female mice and their respective control fed a LF or HF diet (Fig 4).

DIO drastically influenced the expression of GH/IGF-I system in the MFPs of control mice. Specifically, expression of GH-R, IGF-I and IGF-IR, was significantly elevated in both control groups (CORT+/+ and SST+/+). As observed before, GH and PRL expression was again undetectable and no significant changes were observed in IGF-II and PRL-R levels in mammary fat pads of WT groups.

Interestingly, loss of CORT (Fig 4, left-panels) or SST (Fig 4, right-panels) did not significantly influence GH/IGF-I system expression under LF-diet, but clearly impacted the obesity-associated changes. Specifically, CORT-KO mice fed a HF-diet did not show the elevation in GH-R, IGF-I and IGF-IR expression observed in control mice (Fig 4, left-panels). In contrast, loss of SST under obese conditions did not prevent the raise in IGF-IR but significantly blunted the increase in GH-R and IGF-I expression observed in HF-diet control mice (Fig 4; right-panels). In addition, MFPs of HF-fed CORT-KO showed significantly lower levels of PRL-R expression than those of their HF-diet WT counterparts, an observation which was not fully parallel in SST-null mice. Nevertheless, it should be noted that MFPs in both CORT-KO and SST-KO animals exhibited an overall decrease in PRL-R expression due to their genotype (2-way ANOVA analysis, p = 0.0051 and p = 0.0261, in CORT-KO and SST-KO mice, respectively).

### Impact of DIO and loss of SST/CORT on SST/CORT axis in mouse mammary fat pads

**Fig 5:**
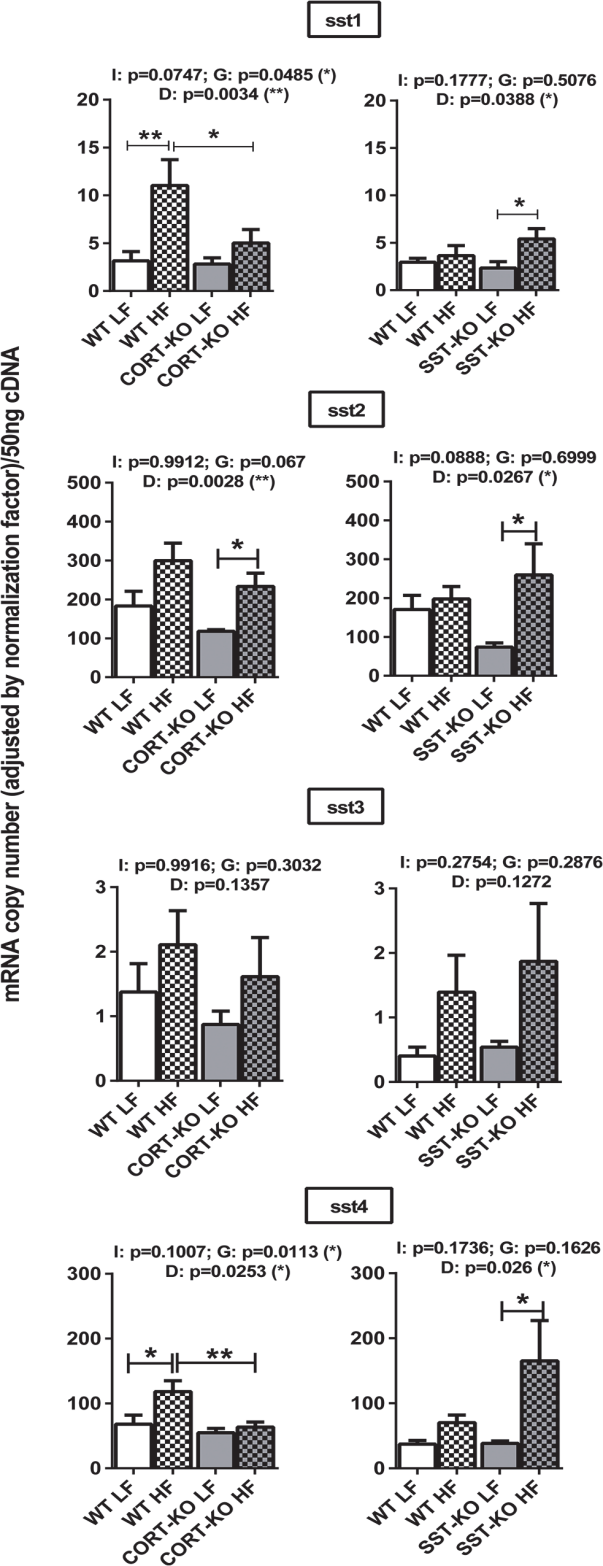
Impact of diet-induced obesity on the expression of SST/CORT/receptor subtypes in the mammary fat pads of CORT-KO, SST-KO and their respective control mice. mRNA levels of sst1, sst2, sst3, sst4 were measured by qPCR. Values represent means ± SEM of the mRNA copy number of each transcript adjusted by the normalization factor (n = 5–6). Global differences by 2-way ANOVA are shown at the top of each graphic (G: genotype effect; D: diet effect I: effect of interaction between genotype and diet; *, p<0.05; **, p<0.01; ***, p<0.001). Asterisks above the bars (*, p<0.05; **, p<0.01) indicate significant differences between groups by Bonferroni post-hoc.

Similar to that found in GH/IGF-I axis, loss of CORT and SST did not significantly alter the expression of the majority of ssts in the MFPs of mice fed with a LF-diet, although we observed that mean of sst2 expression levels in SST-KO LF is 42% with respect to its WT-LF counterpart mice (p = 0.243) (Fig 5). There was an overall effect of the diet in the expression of sst1, sst2 and sst4 as assessed by 2-way ANOVA. A more detailed analysis of this effect revealed an apparent increase of the expression of sst1 and sst4 in HF-fed CORT+/+ mice, while sst1 and sst4 means in HF-fed SST +/+ were 123% (p = 0.995) and 189% (p = 0.995) of the LF-fed, respectively. Likewise, a marked, significant increase was observed for sst1 and sst4 expression in HF-fed SST-KO (Fig 5, right-panel) but not in HF-fed CORT-KO mice (Fig 5, left-panel). Importantly, sst2, which was not significantly elevated by HF-diet in control (+/+) mice, was up-regulated by the lack of CORT and SST. The expression of sst3 was not significantly altered by the diet or the genotype in any group.

### Impact of DIO and loss of SST/CORT on ghrelin axis in mouse mammary fat pads

**Fig 6:**
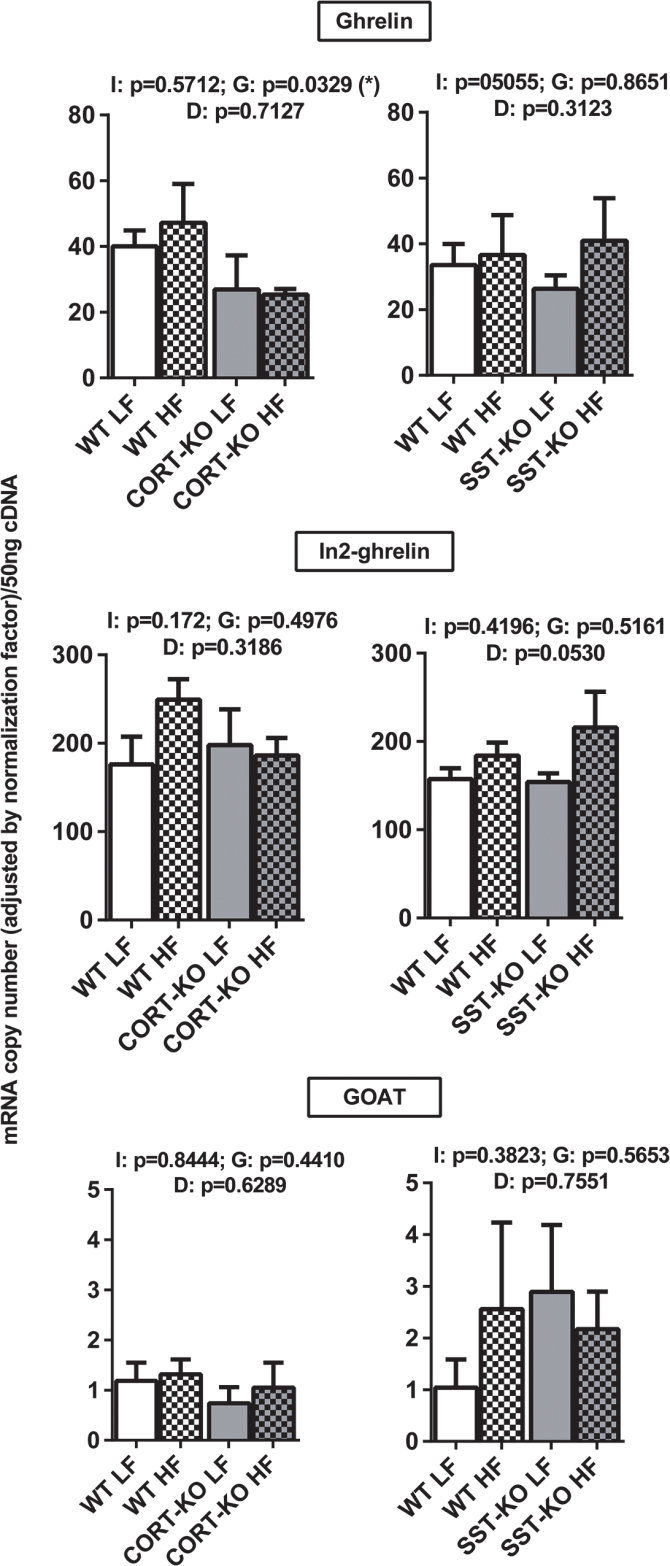
Impact of diet-induced obesity on the expression of ghrelin system in the mammary fat pads of CORT-KO, SST-KO and their respective control mice. mRNA levels of ghrelin, In2-ghrelin, GOAT were measured by qPCR. Values represent means ± SEM of the mRNA copy number of each transcript adjusted by the normalization factor (n = 5–6). Global differences by 2-way ANOVA are shown at the top of each graphic (G: genotype effect; D: diet effect I: effect of interaction between genotype and diet; *, p<0.05).

DIO did not seem to cause major, significant changes in the expression of ghrelin system components (ghrelin, In2-ghrelin and GOAT) in any of the groups analyzed (Fig 6; GHS-R expression was under the detection limit).

## Discussion

This study confirms and extends previous data in human and mice [4–7,52], by showing that some of the components of GH/IGF-I axis are expressed in the mammary fat pads of mature virgin C57Bl/6 female mice. Specifically, IGF-I, IGF-IR, GH-R and PRL-R (which shares a common ancestor with GH-R due to gene duplication) [53] are expressed at high levels in mouse MFPs, while GH (and PRL) expression is barely detectable, at least in mature female mice. These data are consistent with previous studies [4] and reinforce the idea that circulating, rather than locally produced GH acts on MFPs through GH-R to stimulate IGF-I production, which, in turn, can be acting in an autocrine or paracrine fashion to regulate mammary gland development [3,10–14,54,55]. In fact, locally produced IGF-I has been shown to be necessary for mammary gland development [3,10–14] and could be involved in the progression of mammary gland malignancies [10]. In this context, obesity, as a metabolic situation with elevated plasma IGF-I [56], has been associated with a higher risk of developing breast cancer [57,58]. Moreover, since obesity have been shown to markedly alter the expression pattern of GH/IGF-I axis (as well as SST/CORT and ghrelin systems) in other tissues [56,59], we hypothesized that a similar deregulation may also occur in mammary gland, which could therefore influence the (patho)physiology of the mammary gland. Therefore, in order to understand the local changes in the GH/IGF-I axis in response to obesity, in the present study, the expression of the different components of the GH/IGF-I axis was evaluated in the MFPs of diet-induced obese female mice. To the best of our knowledge, this is the first comprehensive characterization of this system in the MFPs of obese females. Importantly, our data show a clear elevation of IGF-I, its receptor and GH-R in the MFPs of diet-induced obese female mice. These results are consistent with previous data showing up-regulation of IGF-IR in the mammary gland of obese women [60] and suggest a mechanism (up-regulation of local expression of the components of the GH/IGF-I axis) which could explain the higher risk of developing mammary malignancies observed in obese individuals [28]. It has to be noted that LFD and HFD are micronutrient-matched diets, and therefore, the changes in transcript expression patterns observed herein should be due to intrinsic DIO-associated alterations (increased dietary fat content and/or fat storage) rather than to the presence/effect of specific diet components. In line with this, and taking into account that obesity is associated to suppressed GH release [56], it seems reasonable to propose that other factors (leptin, insulin, adipokines, inflammatory factors, etc.) should contribute to the increased gene expression of GH/IGF-1 axis components.

SST/CORT and ghrelin systems, which finely regulate GH/IGF-I axis in different tissues [32–35] and may be also involved in mammary gland (patho)physiology [25,29], were also found to be expressed in MFPs. Interestingly, expression of both SST and CORT in mouse MFPs was under the detection limits, which is consistent with previous studies [61] and suggests a marginal or inexistent role for the local production of these ligands in mammary gland physiology. In contrast, the majority of ssts are expressed at detectable levels in the MFPs, where sst2 and sst4 are present at highest levels. Similar expression patterns with sst2 predominance have been observed in human normal and neoplastic [62,63] mammary tissues, confirming the potential role of SST and CORT in regulating MFP physiology. On the other hand, ghrelin and the spliced In2-ghrelin variant were substantially expressed in MFPs, which is consistent with previous reports showing expression of ghrelin [44,45] and In1-ghrelin variant (the human counterpart of mouse In2-ghrelin) [48] in human mammary glands. However, GOAT, the enzyme responsible for ghrelin acylation (described in human mammary gland [64], and GHS-R (not found in mammary tissue [46]) were expressed at very low levels or even under the detection limit.

It has been previously shown that the expression of SST/CORT and ghrelin axes is modulated under metabolic conditions (i.e. obesity) in several tissues [56]. Similarly, in this study we observed an effect of the diet in the expression of sst1, sst2 and sst4, which appeared to be upregulated, perhaps as a compensatory, inhibitory, mechanism in response to GH/IGF-I axis upregulation. Inasmuch as local expression of GH/IGF-I axis is finely regulated by SST and CORT in several tissues [40], we analyzed expression of IGF-I, IGF-IR and GH-R in SST and CORT KO mice under LF- and HF-diets. Surprisingly, lack of SST or CORT did not influence expression of GH/IGF-I components under LFD conditions, despite the fact that circulating GH is elevated in both mouse models and that SST-KO but not CORT-KO mice have increased levels of serum IGF-I, as we have previously reported [50,65]. However, it is noteworthy that lack of SST partially suppressed while lack of CORT completely blunted the up-regulation observed in obese (HF-fed) controls compared to lean (LF-fed) control mice. Our study also provides the first data on the regulation of PRL system components in mammary tissue of CORT-KO and SST-KO mice. Specifically, we observed that PRL-R expression is down-regulated in the absence of CORT and SST, suggesting that, at mammary gland level, CORT and SST can exert a regulatory role on PRL signaling. The significant increase in expression levels of sst2 (the main sst-receptor involved in the inhibitory actions of SST/CORT at many tissues) in MFPs of HF-fed CORT-KO and SST-KO mice could, in part, help to explain the blockade in the obesity-induced up-regulation of GH/IGF-I system observed in these two models as compared with their respective controls (WT-mice). In addition, a compensatory increase in SST levels (circulating and stomach mRNA) observed in CORT-KO mice [50] could provide an explanation to why the obesity-induced up-regulation of GH/IGF-I system is completely blunted only in CORT-KO, since it has been previously reported that SST can directly decrease the expression of GH-R, IGF-I and IGFI-R in a variety of tissues [66–68]. In the current study, constraints in sample availability did not allow us to determine whether changes in mRNA levels observed in the MFPs of these mice models are proportionately translated into functional protein levels, which represents a limitation for our work. This caveat notwithstanding, our findings, when viewed together, clearly support a dissimilar effect of the lack of endogenous SST or CORT in the expression of GH/IGF-I axis components in the mouse MFPs.

In conclusion, our results provide new, original information on key growth factors (GH/IGF-I axis) likely involved in the dysregulation of endocrine/metabolic homeostasis of MFPs in obesity, and indicate that endogenous CORT and SST may be directly involved in the obesity-induced changes observed in GH/IGF-I system in MFPs. In addition, our data also suggest that endogenous CORT is not a simple SST analogue in regulating mammary gland physiology. Altogether, our findings can offer new cues to identify novel molecular targets for diagnosis and/or future treatment of mammary pathologies including breast cancer. Further studies focused on the use of GH-R/IGF-1R antagonists in mouse models developing MG tumors could help to elucidate the possible therapeutic role of these factors in MG pathophysiology.
